# Isolation, Identification, and Functional Characterization of a Rhizosphere Bacterium Promoting the Growth of *Alsophila spinulosa*

**DOI:** 10.3390/microorganisms14051103

**Published:** 2026-05-13

**Authors:** Jiya Wu, Weicheng Yang, Xiaona Zhang, Xianyu Li, Bibo Zhou, Tianyu Liang, Fen Liu

**Affiliations:** 1 School of Life Sciences, Guizhou Normal University, Guiyang 550025, China; 18486593066@163.com (J.W.); 19306513218@163.com (X.L.); 19985972222@163.com (T.L.); 15117707714@163.com (F.L.); 2 Research Center of Buckwheat Industry Technology, College of Life Science, Guizhou Normal University, Guiyang 550025, China

**Keywords:** *Alsophila spinulosa*, *Burkholderia pyrrocinia*, indole-3-acetic acid (IAA), IAA biosynthetic pathways, antioxidant enzymes

## Abstract

*Alsophila spinulosa* is a tree fern designated as a second-class nationally protected species in China and valued for its medicinal and ornamental properties. Its slow growth and susceptibility to environmental stresses pose challenges to its cultivation. Plant-growth-promoting rhizobacteria (PGPR) can enhance plant development by producing phytohormones, such as indole-3-acetic acid (IAA). In this study, 39 IAA-producing strains were isolated from the rhizosphere of *A. spinulosa.* Morphological and molecular analyses identified the highest IAA-producing strain, R74, as *Burkholderia pyrrocinia*. Its optimal inoculum age was determined to be 12–20 h, and its optimal culture conditions for IAA production were 24 h of incubation, 32 °C and pH 7.0. Whole-genome sequencing revealed that the genome of strain R74 is 8,347,169 bp in size with a GC content of 67%, comprising 7543 genetic elements. Further genomic analysis showed that IAA biosynthesis in R74 involves the tryptophan side-chain oxidase (TSO) pathway and the tryptophan-independent pathway. Pot experiments revealed that inoculation with R74 increased the height, root length, stem diameter, and biomass of *A. spinulosa* seedlings. It also increased antioxidant enzyme activities, elevated soluble protein and chlorophyll contents, and reduced malondialdehyde levels. This study provides an empirical basis for the development of *Burkholderia*-based biofertilizers to promote *A. spinulosa* growth.

## 1. Introduction

*Alsophila spinulosa*, a tree fern belonging to the family Cyatheaceae and genus *Alsophila*, is an ancient relict species originating in the Mesozoic era and is widely regarded as a “living fossil” among ferns. In China, Cyatheaceae species have experienced continuous endangerment due to habitat fragmentation and are currently under state protection, with all species within this family now classified as second-class nationally protected plants. Various flavonoids and phenolic compounds have been isolated from the stems of *A. spinulosa* and have been utilized in traditional medicine [1]. In addition to its medicinal value [2], *A.spinulosa* plays a crucial role in studies of speciation, phytogeography, paleobotany, and paleoenvironmental change [3]. Although *A. spinulosa* can be locally dominant in the tree stratum, it remains severely impacted by anthropogenic disturbances in the shrub and seedling layers. Notably, its survival rate drops below 1% by the seventh age class (half-mature stage) [4,5]. Conventional conservation approaches, such as tissue culture [6,7], ex situ transplantation [8,9], and habitat restoration [10,11], are often constrained by high costs, low survival rates, and potential secondary ecological damage [12]. Consequently, there is a growing shift toward safer, more sustainable alternatives, including microbe-based strategies [3,13].

Plant growth-promoting rhizobacteria (PGPR) are beneficial microorganisms that inhabit the rhizosphere of plants. These rhizobacteria directly or indirectly promote plant growth by producing essential phytohormones or converting insoluble mineral compounds into bioavailable forms [14,15,16]. They exert growth-promoting effects via multiple mechanisms, including the secretion of indole-3-acetic acid (IAA), cytokinins, gibberellins, and ACC deaminase, as well as phosphate solubilization and siderophore production [17,18,19,20]. Currently, well-characterized PGPR belong primarily to the genera *Bacillus*, *Pseudomonas*, and *Burkholderia*. *Bacillus* spp. actively colonize the rhizosphere and enhance plant growth through phytohormone production, phosphate solubilization, and nitrogen fixation [21,22,23]. For example, *Bacillus amyloliquefaciens* GB03 promotes growth and boosts abiotic stress tolerance in *Arabidopsis thaliana* [24]. *Pseudomonas* spp. are widely recognized for their ability to promote plant growth at the molecular level, benefiting both root and shoot development while conferring additional advantages to host plants [25]. Notably, *Pseudomonas putida* AKMP7 enhances thermotolerance in wheat seedlings and improves drought resistance in rice [26,27]. *Burkholderia* spp. are commonly found in soil, particularly in the rhizosphere of grasses [28,29]. *Burkholderia pyrrocinia* JK-SH007 has been shown to promote poplar growth and effectively control canker disease [30,31]. Despite these, research on PGPR associated with the endangered tree fern *A. spinulosa* remains scarce. Identifying effective PGPR strains for this species could substantially contribute to its conservation and restoration [12,32].

In this study, we isolated and identified rhizosphere bacteria from *A. spinulosa*, characterized their plant growth-promoting traits, and evaluated their effects on *A. spinulosa* growth. Whole-genome sequencing was performed to analyze the biosynthetic pathways associated with key growth-promoting genes. Through this integrative approach, we aimed to preliminarily screen for rhizobacteria with superior growth-promoting efficacy on *A. spinulosa* and to establish a theoretical foundation for future research addressing both rapid growth and effective conservation of this endangered fern species.

## 2. Materials and Methods

### 2.1. Culture Media Preparation

Luria–Bertani (LB) solid culture medium: Tryptone 10.0 g, Yeast extract 5.0 g, NaCl 10.0 g, Agar 20 g, Distilled water 1000 mL, pH 7.0. LB liquid culture medium: Tryptone 10.0 g, Yeast extract 5.0 g, NaCl 10.0 g, Distilled water 1000 mL, pH 7.0 [33].

### 2.2. Collection and Isolation of Rhizosphere Bacterial Strains

#### 2.2.1. Soil Sampling

Rhizosphere soil samples were collected from healthy *Alsophila spinulosa* plants in the Zunyi National *Alsophila spinulosa* Nature Reserve (28°39′–28°47′ N, 105°96′–106°97′ E) using the five-point sampling method. The samples were immediately transported to the laboratory and stored at 4 °C prior to further processing.

#### 2.2.2. Enrichment and Isolation of Bacterial Strains

Fresh rhizosphere soil (10 g) was suspended in 90 mL of sterile water and shaken at 30 °C and 180 rpm for 2 h in an orbital shaker (THZ-C, Taicang Dazhong Experimental Instrument Co., Ltd., Taicang, China) to obtain a 10^−1^ suspension. The suspension was serially diluted to concentrations ranging from 10^−2^ to 10^−5^. For each dilution, a 100 μL aliquot was spread-plated onto LB solid medium in triplicate. The plates were incubated inverted at 30 °C for 72 h in a constant temperature incubator (SPX-280 manufactured by Ningbo Jiangnan Instrument Factory, Ningbo, China), and colony morphology was observed periodically. Single colonies with distinct morphologies were selected and streaked onto freshly prepared LB solid medium using a sterile inoculation loop. This streak-isolation purification procedure was repeated 3–4 times until pure cultures were obtained.

### 2.3. Purification and Preservation of Bacterial Strains

For each purified strain, a single colony was inoculated into LB liquid medium and incubated at 30 °C with shaking at 180 rpm for 24 h. Following incubation, the cultures were mixed with an equal volume of sterile 30% (*v*/*v*) glycerol solution and aliquoted into sterile 1.5 mL micro-centrifuge tubes (Beijing Labgic Technology Co., Ltd., Beijing, China). The samples were labeled and stored at 4 °C for preservation.

### 2.4. Determination of IAA Production by Soil Bacterial Strains

To identify the strain with the highest IAA production, the IAA yields of all purified strains were measured.

(1) Preliminary Screening of IAA-Producing Strains: The bacterial strains preserved in Section 2.3 were reactivated on LB solid medium to restore viability. A single colony of each strain was inoculated into 4 mL of LB liquid medium supplemented with 100 mg/L L-tryptophan (Phygene Biotechnology Co., Ltd., Fuzhou, China) and incubated at 30 °C with shaking at 180 rpm for 24 h. For each strain, an aliquot of 200 μL of the bacterial culture was mixed with an equal volume of Salkowski colorimetric reagent (Feijie Biotechnology Co., Ltd., Shanghai, China) and incubated in the dark for 30 min to allow color development. LB medium supplemented with 100 mg/L L-tryptophan served as the negative control, while an IAA standard solution served as the positive control. A color change to red indicated the ability of the strain to produce IAA, with a deeper color suggesting higher IAA production [34,35,36].
(2) IAA Standard Curve Preparation: An IAA standard stock solution (100 mg/L) was prepared by dissolving 100 mg of IAA standard (Macklin, Shandong Keyuan Biochemical Co., Ltd., Heze, China) in a minimal volume of ethanol and diluting to 100 mL with deionized water. A series of IAA standard solutions at concentrations of 0, 5, 10, 15, 20, 25, 30, and 35 mg/L was prepared and stored in the dark [37]. An aliquot (1 mL) of each standard was mixed with an equal volume of Salkowski colorimetric reagent and incubated at room temperature in the dark for 30 min. Absorbance was measured at 530 nm using a microplate reader (K6600A, Beijing Kai’ao Technology Development Co., Ltd., Beijing, China), with the IAA standard solution serving as a positive control. The standard curve was generated by plotting absorbance against IAA concentration [38].
(3) IAA Quantification: Bacterial strains showing IAA-producing activity in the preliminary screening were quantitatively assessed in LB medium supplemented with 100 mg/L L-tryptophan. Cultures were grown under the same conditions described in Section 2.3. Bacterial growth was monitored by measuring OD_600_ using a microplate reader. The culture was centrifuged at 8000 rpm for 10 min (TGL-20M, Shanghai Luxiangyi Centrifuge Instrument Co., Ltd., Shanghai, China) [39]. 1 mL of the supernatant was mixed with an equal volume of Salkowski colorimetric reagent in a quartz cuvette and incubated in the dark for 30 min. Color development was observed, and absorbance was measured at 530 nm. IAA concentration was determined using the standard curve. Values were normalized to cell density (OD_600_) to calculate IAA production per unit biomass (OD_600_ = 1) [40].
(4) HPLC Confirmation of IAA Production: Fermentation broth of the activated strain R74 was submitted to Beijing Hangjian Detection Service Co., Ltd. (Beijing, China) for high-performance liquid chromatography (HPLC) analysis. The specific procedures are as follows: An IAA standard stock solution was prepared by dissolving the standard in methanol, followed by serial dilution to obtain working standard solutions at concentrations of 5, 10, 20, 50, and 100 mg/L. All solutions were filtered through 0.22-μm organic membranes prior to injection. A standard curve was constructed by plotting peak area against concentration. Chromatographic conditions were as follows: mobile phase of methanol and 0.2% acetic acid in water (55:45, *v*/*v*); flow rate of 0.6 mL/min; column temperature of 30 °C; detection wavelength of 280 nm; and injection volume of 10 μL. IAA concentration was quantified by the external standard method using the sample peak area and the standard curve [41].
(5) Cultivation of the strain with the highest IAA-producing strain: The strain exhibiting the highest IAA production was inoculated into 100 mL of LB liquid medium and cultured overnight at 32 °C in an orbital shaker at 180 rpm. Upon reaching the early exponential growth phase (OD_600_ = 0.6−0.8), the bacterial cells were harvested by centrifugation at 8000 rpm for 10 min. The cell density was then determined, and the strain was designated as R74.

### 2.5. Morphological and Molecular Biological Identification of Strain R74

Morphological Observation: Strain R74 was streaked onto LB solid medium and incubated inverted at 30 °C until single colonies appeared. The colony morphology, including size, shape, and pigmentation, was then examined. Gram staining was performed using a kit provided by Changde Man Biotechnology Co., Ltd. (Changde, China), following the manufacturer’s instructions.

Scanning Electron Microscopy (SEM) Analysis: Strain R74 was submitted to Wuhan Servicebio Technology Co., Ltd., Wuhan, China, for scanning electron microscopy analysis. The following procedures were performed by the company: Bacterial cells were collected by centrifugation, rinsed with phosphate-buffered saline (PBS), and fixed with electron microscopy fixative. After rinsing with 0.1 M phosphate buffer (pH 7.4), the cells were post-fixed with 1% osmium tetroxide in the dark at room temperature for 1–2 h, followed by another rinse. The samples were then dehydrated through a graded ethanol series (30–100%) and treated with isoamyl acetate. Following critical point drying, the specimens were sputter-coated with gold for 30 s and examined using a scanning electron microscope, with images captured for analysis.

DNA Extraction and Sequencing: The cultured bacterial strain R74 was submitted to Sangon Biotech (Shanghai) Co., Ltd. (Shanghai, China), for sequencing. The following procedures were performed by the company: Genomic DNA was extracted using the NucleoBond^®^ HMW DNA kit (MN NucleoBond, Düren, Germany, 740160.20). DNA concentration and purity were determined via Qubit 4.0 (Thermo, Q33226, Waltham, MA, USA) and Nanodrop (SMA4000, Taipei, Taiwan, China). DNA integrity was assessed by 0.75% agarose gel electrophoresis. The genomic DNA was then divided into two parts. One part was randomly fragmented to construct a library with a 300 bp insert size. This library was sequenced on the MGI DNBSEQ-T7 platform using a paired-end 150 bp sequencing strategy [42]. The other part was directly subjected to end-repair, 3′ adenylation, adapter ligation, and motor protein ligation. The product was purified using Agencourt AMPure XP Beads (Beckman, A63881, Brea, CA, USA). Finally, fragments larger than 1 kb were selected for single-molecule nanopore DNA sequencing on MinION Flow Cell (ONT, R9.4.1) [43].

Phylogenomic Tree Construction: The 16S rRNA sequence of this strain was compared against the NCBI 16S rRNA database using BLASTn (v 2.2.28), and the top 30 strains with the highest similarity were selected. Roary software (v3.13.0) was used to perform pan-genome analysis on the complete genome sequences of this strain and its closely related strains, identifying all single-copy core genes shared among the strains [44]. The selected core gene sequences were concatenated, and multiple sequence alignment and trimming were performed using MAFFT (v7.49) [45]. Based on the alignment of single-copy core genes, a core gene phylogenetic tree was constructed using FastTree (v 2.1.7) with the neighbor-joining (NJ) method [46]. The analysis workflow for the core gene set followed the standard Roary protocol (https://github.com/sanger-pathogens/Roary, accessed on 5 September 2025).

### 2.6. Comparative Genome Analysis

The average nucleotide identity (ANI) of closely related strains was calculated using fastANI [47]. Species assignment followed the established threshold in microbial taxonomy, where an ANI value of ≥95% was considered to represent the same species. All reference genomes were annotated with NCBI accession numbers (GCA) to ensure sequence traceability.

### 2.7. Growth Curve Determination of Strain R74

To revive the cryopreserved strain, a single colony of strain R74 was picked from LB solid medium using an inoculation loop, inoculated into 50 mL of LB liquid medium, and incubated at 30 °C with shaking at 180 rpm for 24 h. An aliquot (200 μL) of this seed culture was transferred into 100 mL of fresh LB liquid medium and incubated at 30 °C with shaking at 180 rpm for 32 h in an orbital shaker. Bacterial growth was monitored by measuring the optical density at 600 nm (OD_600_) at 4 h intervals over the 32 h period, yielding eight time points. All measurements were performed in biological triplicate. The growth curve was plotted to identify the time point corresponding to the highest bacterial activity [48].

### 2.8. Optimization of IAA Production by Strain R74

Aliquots (100 μL) of strain R74 suspension were inoculated into 2 mL of LB liquid medium supplemented with 100 mg/L L-tryptophan (Phygene Biotechnology Co., Ltd., Fuzhou, China). The cultures were incubated at 30 °C with shaking at 180 rpm. To determine IAA production, samples were collected at 16, 20, 24, 28, 30, and 32 h. To determine the optimal temperature, cultures were incubated at 24, 28, 30, 32, 34, and 36 °C with shaking at 180 rpm for 24 h, and IAA concentrations were measured. To determine the optimal pH, the medium pH was adjusted to 5.5, 6.0, 6.5, 7.0, 7.5, and 8.0, and cultures were incubated at 32 °C with shaking at 180 rpm for 24 h, followed by IAA quantification.

### 2.9. Genome Assembly and Annotation

Short reads were filtered using Fastp (v0.23.0), and long reads were filtered using Fastplong (v0.2.2) by removing adapters and low-quality reads. Genome assembly was performed using Unicycler (v0.5.1) with default parameters [49], followed by proofreading with NextPolish (v1.4.1). After assembly, BUSCO (v4.1.4) was used to assess the completeness of conserved core genes [50], and CheckM (v1.0.12) was used to evaluate genome completeness and contamination levels. Subsequently, based on the assembly results, coding sequences (CDS), tRNA, and rRNA were predicted using NCBI PGAP, and tandem repeat DNA motifs were identified using TRF (v4.09) [51]. All above analyses were performed by Sangon Biotech (Shanghai) Co., Ltd. The whole genome sequence of strain R74 was deposited into GenBank with the accession number GCA_056783395.1.

### 2.10. Analysis of IAA Production-Related Genes in Strain R74

The protein sequences of genes were aligned against multiple databases including CDD, KOG, COG, NR, NT, PFAM, Swissprot, and TrEMBL using NCBI Blast+ to obtain functional annotation information [52]. Gene ontology (GO) functional annotation was acquired based on the annotation results from Swissprot and TrEMBL. The KEGG annotation of genes was performed via KAAS [53]. Candidate genes involved in auxin biosynthesis were screened according to the KEGG annotation results.

### 2.11. Determination of Growth Indices of Alsophila spinulosa Inoculated with Burkholderia pyrrocinia R74

Pot experiments were conducted at the greenhouse experimental base of Guizhou Normal University (26°39′ N, 106°63′ E). *A. spinulosa* seedlings obtained via plant tissue culture were used as the plant material [54]. The seedlings were pre-cultivated in nursery pots and acclimatized in a controlled-environment chamber. After hardening, seedlings with comparable growth were selected and transplanted into pots. The plant material consisted of one-year-old acclimatized seedlings with uniform growth, a height of 12 ± 1 cm, and four to five fully developed pinnate compound leaves. All seedlings were free from pests and diseases and exhibited uniform physiological status. To ensure consistent experimental conditions, pots of uniform size were used (external diameter: 17.5 cm, internal diameter: 14.5 cm, height: 12.5 cm), each containing 1.7 kg of soil mixture (red soil:humus:peat = 2:1:1, *v*/*v*/*v*) as a suitable transplanting substrate for *A. spinulosa* tissue-cultured seedlings [7]. Prior to the experiment, the soil was sieved and subsequently autoclave-sterilized at 121 °C for 30 min.

Seedlings with uniform growth were selected after one month of establishment. For the treatment group, the bacterial pellet was washed three times with sterile water and resuspended to obtain a bacterial suspension at a concentration of 1 × 10^7^ CFU/mL. 5 mL of the suspension was then applied to the root zone of each potted *A. spinulosa* seedling. The control group received an equal volume of sterile water. Each treatment consisted of 30 biological replicates, with one plant per pot. The pots were arranged in rows of 10 plants, and the experiment was conducted in triplicate. All seedlings were arranged in a completely randomized design within the greenhouse, and pot positions were rotated weekly to minimize positional effects. Environmental conditions were maintained consistently between the treatment and control groups. At 15, 30, and 60 days post-inoculation, seedlings from both groups were collected, and plant height, stem diameter, and root length were measured in the laboratory.

#### 2.11.1. Determination of Chlorophyll Content in *Alsophila spinulosa* Seedlings

Fresh leaves (0.5 g) were collected from well-grown *A. spinulosa* seedlings in both the experimental and control groups at 15, 30, and 60 days, with three replicates per group. The leaf samples were placed in a mortar and ground thoroughly with a small volume of 95% ethanol, quartz sand (to facilitate grinding), and calcium carbonate (to prevent chlorophyll degradation). The homogenate was filtered through filter paper, and the filtrate was collected and diluted to a final volume of 100 mL with 95% ethanol. The absorbance of the solution was measured at OD_645_ and OD_663_ using a microplate reader. The obtained values were substituted into the following formulas to calculate the concentrations of chlorophyll a (Chl a), chlorophyll b (Chl b), and total chlorophyll (Chl T) [55]. Chlorophyll calculation formulas:

Ca (mg/L) = 12.7 OD_663_ − 2.69 OD_645_

Cb (mg/L) = 22.9 OD_645_ − 4.68 OD_663_

CT (mg/L) = Ca + Cb = 20.2 OD_645_ + 8.02 OD_663_

#### 2.11.2. Determination of Antioxidant Enzyme Activities and Malondialdehyde Content in *Alsophila spinulosa* Seedlings

Leaf samples were collected from well-grown *Alsophila spinulosa* seedlings in both the experimental and control groups at 15, 30, and 60 days, with three replicates per group. The content of malondialdehyde (MDA) and the activities of antioxidant enzymes including superoxide dismutase (SOD), peroxidase (POD), and catalase (CAT) were assayed using commercial detection kits (Grace Biotechnology Co., Ltd., Suzhou, China) [56]. Detailed procedures were as follows:

Determination of Malondialdehyde (MDA): Fresh leaf tissue (0.5 g) was homogenized with 1 mL of extraction buffer in an ice bath. The homogenate was centrifuged at 12,000 rpm for 10 min at 4 °C, and the supernatant was collected and kept on ice until analysis. The microplate reader was preheated for 30 min, and a water bath was heated to 90–95 °C. The following components were sequentially added to an Eppendorf tube: 300 μL of working solution and 200 μL of sample. After thorough mixing, the tube was sealed with sealing film and incubated in a 90–95 °C water bath for 30 min. The tube was then removed and cooled on ice, followed by centrifugation at 12,000 rpm for 10 min at 25 °C. An aliquot (200 μL) of the supernatant was transferred to a 96-well plate, and absorbance was measured at 532 nm (OD_532_) and 600 nm (OD_600_). The absorbance difference was calculated as ΔA = A_532_ − A_600_.

MDA content (nmol/g fresh weight) was calculated as: 32.2 × ΔA/W, where W represents the fresh weight (g) of the sample.

Determination of Peroxidase (POD) Activity: Fresh leaf tissue (0.25 g) was homogenized with 1 mL of extraction buffer in an ice bath. The homogenate was centrifuged at 12,000 rpm for 10 min at 4 °C, and the supernatant was collected and kept on ice until analysis. The microplate reader was preheated for 30 min, and the wavelength was set to 470 nm (OD_470_). Reagents 1, 2, and 3 were equilibrated to room temperature (25 °C) prior to measurement. The following components were sequentially added to a 96-well plate: 10 μL of sample, 40 μL of Reagent 1, 140 μL of Reagent 2, and 10 μL of Reagent 3. After thorough mixing, absorbance at 470 nm was immediately recorded as A1, and again after 1 min as A_2_. The absorbance difference was calculated as ΔA = A_2_ − A_1_.

One unit (U) of POD activity was defined as the amount of enzyme that causes an increase of 1.0 in absorbance at 470 nm (OD_470_) per minute per gram of fresh tissue.

POD activity (ΔOD_470_/min/g fresh weight) was calculated as: 100 × ΔA/W × D, where W represents the fresh weight (g) of the sample, and D represents the dilution factor (D = 1 for undiluted samples).

Determination of Catalase (CAT) Activity: Fresh leaf tissue (0.5 g) was homogenized with 1 mL of extraction buffer in an ice bath. The homogenate was centrifuged at 12,000 rpm for 10 min at 4 °C, and the supernatant was collected and kept on ice until analysis. The microplate reader was preheated for 30 min, and the wavelength was set to 510 nm (OD_510_). Reagent 2 was prepared as follows: after flicking the tube to ensure the reagents settled at the bottom, 80 μL was transferred to two new Eppendorf tubes, and 1.56 mL of distilled water was added to each tube and mixed thoroughly. For the blank control (performed once): 80 μL of Reagent 1, 20 μL of Reagent 2, and 100 μL of Reagent 3 were immediately mixed, and 10 μL of this mixture was promptly subjected to colorimetric reaction following the sequential addition protocol. The absorbance value was recorded as A_blank_ (note: if multiple samples were analyzed simultaneously, they could be processed in batches as the reaction time was 5 min).

The following components were sequentially added to an Eppendorf tube: 10 μL of sample, 70 μL of Reagent 1, and 20 μL of Reagent 2. The mixture was thoroughly mixed (bubble formation indicated enzyme activity; more bubbles indicated higher activity) and allowed to react at room temperature (25 °C) for exactly 5 min. Subsequently, 100 μL of Reagent 3 was added and mixed. Immediately afterward, 10 μL of this mixture was collected. If turbidity was observed, the mixture was centrifuged at 8000 rpm for 10 min at room temperature or 4 °C, and the supernatant was used for the subsequent colorimetric reaction.

For the colorimetric reaction, 10 μL of the mixture, 900 μL of Reagent 1, and 290 μL of Reagent 4 were mixed thoroughly and incubated at room temperature (25 °C) for 5 min. An aliquot (200 μL) was transferred to a 96-well plate, and absorbance was measured at 510 nm. The absorbance difference was calculated as ΔA = A_blank_ − A_sample_.

One unit (U) of CAT activity was defined as the amount of enzyme that catalyzes the decomposition of 1 μmol of H_2_O_2_ per minute per gram of fresh tissue at 25 °C.

CAT activity (μmol/min/g fresh weight) was calculated as: 141.6 × (ΔA + 0.0137)/W × D, where W represents the sample weight (g) of the sample, and D represents the dilution factor (D = 1 for undiluted samples).

Determination of Superoxide Dismutase (SOD) Activity: Fresh leaf tissue (0.25 g) was homogenized with 1 mL of extraction buffer in an ice bath at 4 °C. The homogenate was centrifuged at 12,000 rpm for 10 min at 4 °C, and the supernatant was collected as the test solution. The microplate reader was preheated for 30 min, and the wavelength was set to 450 nm (OD_450_). Reagents 1, 3, and 4 were incubated in a 25 °C water bath for at least 5 min prior to measurement. A multichannel pipette was used to minimize errors caused by timing differences in reagent addition across wells. Reagent 4 was thoroughly mixed before each addition to ensure homogeneity. The following components were sequentially added to a 96-well plate: Sample well: 70 μL Reagent 1, 20 μL Reagent 2, 20 μL sample, 10 μL Reagent 3, and 80 μL Reagent 4. Blank control well 1: 70 μL Reagent 1, 20 μL Reagent 2, 20 μL distilled water, 10 μL Reagent 3, and 80 μL Reagent 4. Blank control well 2: 70 μL Reagent 1, 40 μL distilled water, 10 μL Reagent 3, and 80 μL Reagent 4. After thorough mixing, the plate was incubated at room temperature (25 °C) in the dark for 30 min. Absorbance of each well was measured at 450 nm (OD_450_).

The inhibition percentage was calculated as: [(A_blank1_ − A_blank2_) − (A_sample_ − A_sample-control_)]/(A_blank1_ − A_blank2_) × 100%.

One unit of SOD activity was defined as the amount of enzyme required to achieve 50% inhibition in the xanthine oxidase-linked reaction system.

SOD activity (U/g fresh weight) was calculated as: 10 × inhibition percentage ÷ (1 − inhibition percentage)/W × D, where W represents the sample weight (g) of the sample, and D represents the dilution factor (D = 1 for undiluted samples).

Determination of Soluble Protein Content by the Bradford Method: Fresh leaf tissue (0.5 g) was homogenized with 1 mL of extraction buffer in an ice bath. The homogenate was centrifuged at 12,000 rpm for 10 min at 4 °C, and the supernatant was collected and kept on ice until analysis. The microplate reader was preheated for 30 min, and the wavelength was set to 600 nm. The following components were added to a 96-well plate: Sample well: 40 μL of test solution and 200 μL of Reagent 1; Blank well: 40 μL of distilled water and 200 μL of Reagent 1. After thorough mixing, the plate was incubated at room temperature (25 °C) for 10 min. The absorbance was measured at 600 nm (OD_600_), with colorimetric reading completed within 5–15 min. The absorbance difference was calculated as ΔA = A_sample-well_ − A_blank-well_.

The standard curve was defined as: y = 3.8457x + 0.0136, where x represents the standard concentration (mg/mL) and y represents ΔA.

Protein content (mg/g fresh weight) was calculated as: 0.26 × (ΔA − 0.0136) × D/W, where W represents the sample weight (g) of the sample, and D represents the dilution factor (D = 1 for undiluted samples).

### 2.12. Statistical Analysis

All experimental data were presented as the mean ± standard deviation (SD) of three independent replicates. Statistical analyses were performed using SPSS version 26.0, including independent samples *t*-tests and one-way analysis of variance (ANOVA). Duncan’s multiple range test was used for post hoc comparisons, with differences considered significant at *p* < 0.05 and highly significant at *p* < 0.01.

## 3. Results

### 3.1. Determination of IAA Content Secreted by the Strain

A standard curve for IAA quantification was generated by plotting absorbance at OD_530_ against IAA concentration. Linear regression analysis yielded the equation y = 0.0092x + 0.0833, with a coefficient of determination R^2^ = 0.993 (Supplementary Figure S1). Qualitative assessment with Salkowski reagent showed that 39 isolates developed pink coloration of varying intensities, indicating their ability to produce IAA. Quantitative measurements revealed considerable variation in IAA production levels among the strains (Table 1). Strain R74 exhibited the highest IAA production, reaching 53.492 mg/(L·OD_600_), followed by strain R3 with 40.010 mg/(L·OD_600_). Strain R83 showed the lowest production at 7.330 mg/(L·OD_600_). Therefore, strain R74, with the highest IAA-producing capacity, was selected for subsequent experiments.

### 3.2. Validation of IAA Production by High-Performance Liquid Chromatography (HPLC)

**Figure 1:**
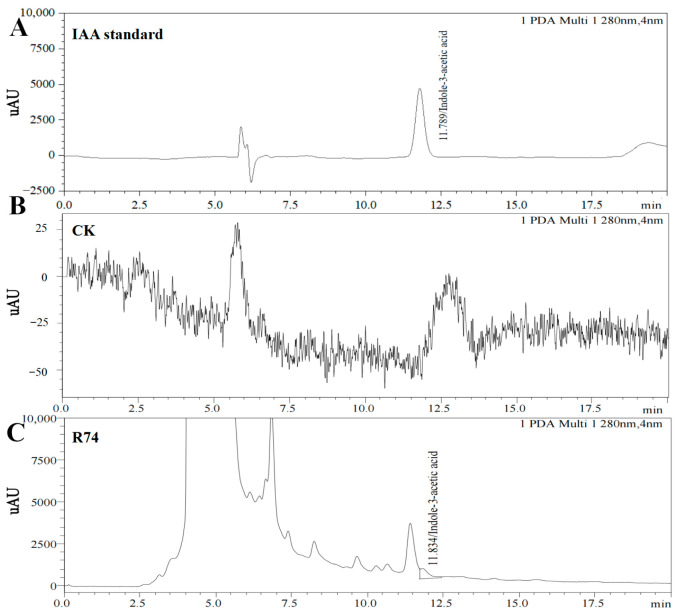
High-performance liquid chromatography (HPLC) profiles of indole-3-acetic acid (IAA) produced by strain R74. IAA standard, authentic IAA; CK, blank control; R74, fermentation broth of strain R74.

High-performance liquid chromatography was employed to verify IAA production by strain R74 (Figure 1). The IAA standard showed a characteristic absorption peak at 11.789 min, with a standard curve defined as f(x) = 2.56810 × 10^−5^x − 0.747050 (R^2^ = 0.9997), indicating excellent linearity. A corresponding target peak was observed at 11.834 min in the fermentation broth of strain R74, whereas no such peak was detected in the blank control, which confirmed the identity of IAA. Quantification by the external standard method yielded an IAA concentration of 0.319 mg/L in the fermentation broth, providing chromatographic evidence that *B. pyrrocinia* R74 possesses the capacity for IAA biosynthesis.

### 3.3. Morphological and Molecular Characteristics of Strain R74

**Figure 2:**
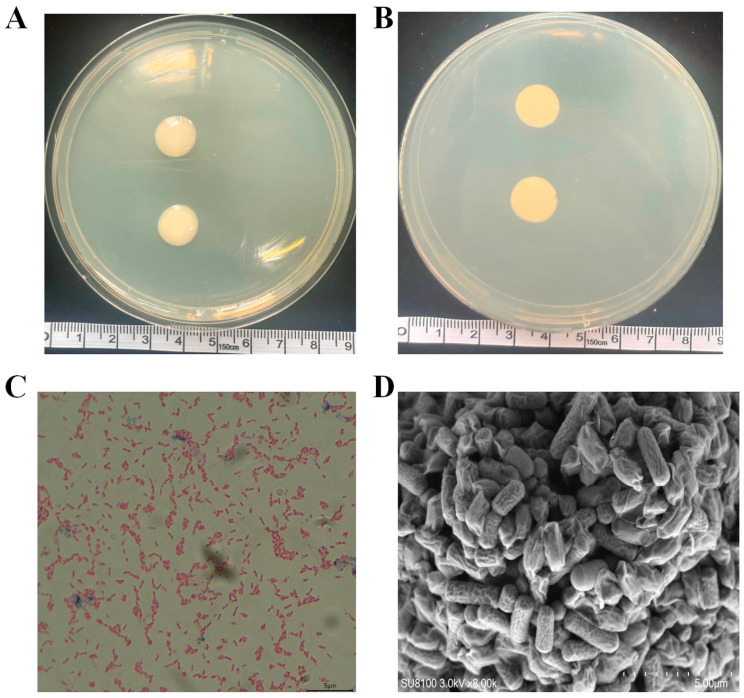
Morphological characteristics of strain R74: (**A**) front view of R74 colony cultured for 72 h; (**B**) back view of R74 colony cultured for 72 h; (**C**) Gram staining of strain R74, magnification 100×, scale bar = 5 μm; (**D**) scanning electron microscopy (SEM) observation of strain R74 morphology, scale bar = 5 μm.

After 72 h of incubation at 28 °C on LB solid medium, colonies of strain R74 were circular, 0.4–0.6 cm in diameter, with a milky white, moist, glistening surface and entire margins. Colonies exhibited a distinct hemispherical elevation without spreading growth or surface wrinkles (Figure 2A). The reverse side of colonies displayed a uniform light-yellow pigmentation, with no diffusing halo (Figure 2B). Gram staining revealed the cells to be Gram-negative bacteria (Figure 2C). Scanning electron microscopy (SEM) showed short rods measuring 0.8–1.2 µm in width and 2.0–2.5 µm in length, occurring singly or in pairs (Figure 2D). These morphological characteristics align with those of *Burkholderia pyrrocinia*, providing preliminary evidence for species identification.

### 3.4. Comparative Genomics and Phylogenetic Analysis

**Figure 3:**
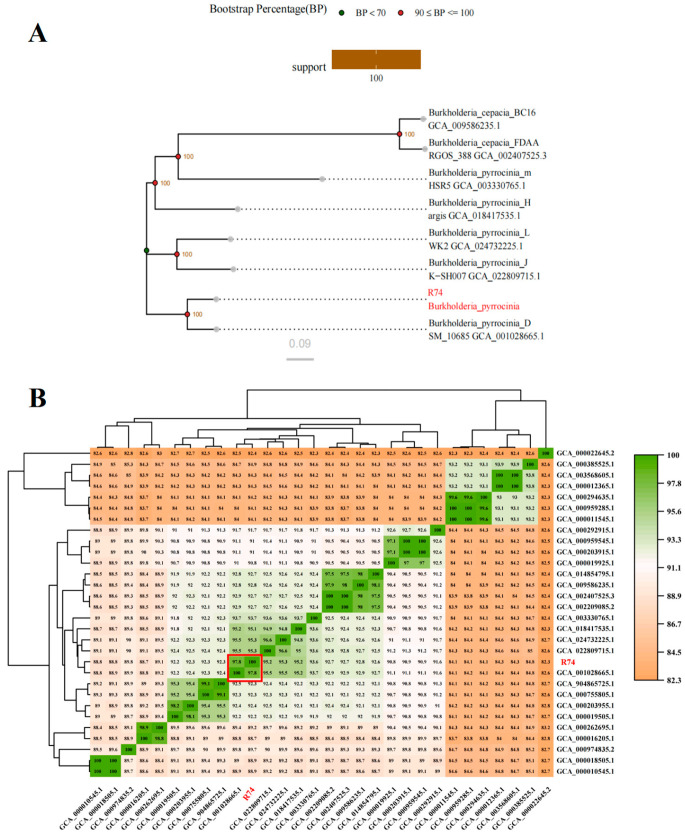
(**A**) Phylogenetic tree constructed using the neighbor-joining (NJ) method based on single-copy core genes. The scale bar (0.09) denotes the genetic distance, with branch lengths indicating 9 substitutions per 100 nucleotide positions; (**B**) Heatmap of average nucleotide identity (ANI) between strain R74 and 29 other strains. Pairwise ANI similarity is represented by a color gradient, with darker colors indicating higher similarity.

Phylogenomic analysis based on single-copy core genes placed strain R74 in the same clade as the *B. pyrrocinia* type strain, with a bootstrap support of 100% (Figure 3A). ANI values between strain R74 and *B. pyrrocinia* reference strains were ≥95% (Figure 3B), meeting the prokaryotic species delineation threshold. Together, morphological and genomic evidence confirmed strain R74 as *Burkholderia pyrrocinia*.

### 3.5. Growth Curve of Burkholderia pyrrocinia R74

**Figure 4:**
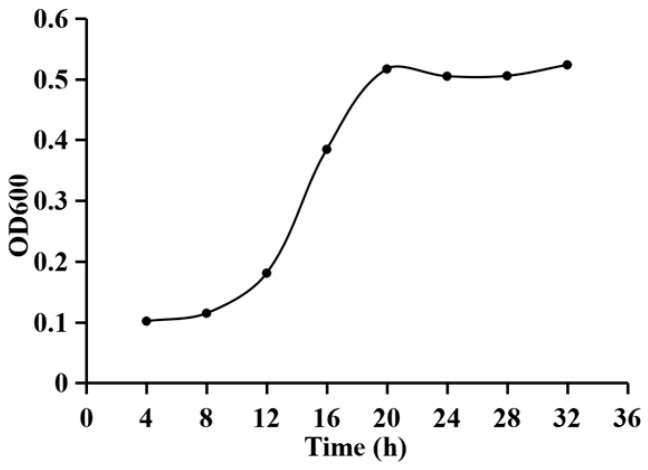
Growth curve of *Burkholderia pyrrocinia* R74.

The growth curve of *B. pyrrocinia* R74 is shown in Figure 4. The strain exhibited a lag phase from 1 to 8 h, followed by an exponential phase from 8 to 20 h, and entered the stationary phase after 20 h. Based on these growth characteristics, the optimal inoculum age for R74 was determined to be 12–20 h, corresponding to the mid-to-late exponential phase.

### 3.6. Optimization of Culture Conditions for IAA Production by Strain R74

**Figure 5:**
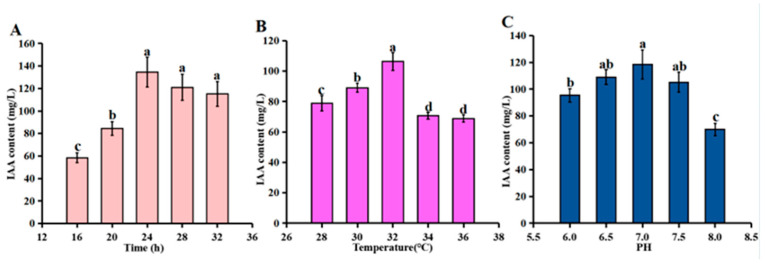
Effect of incubation time, temperature and pH on IAA secretion by R74. Values are mean ± SD (n = 3). Different letters indicate significant differences (ANOVA with Duncan’s test, *p* < 0.05). (**A**) IAA production ability of strain R74 over time. (**B**) IAA content of strain R74 under varying temperature conditions. (**C**) IAA content of strain R74 under varying pH conditions.

IAA production by strain R74 increased with culture time, peaking at 24 h, followed by a plateau (Figure 5A). Within the temperature range of 28–32 °C, IAA production exhibited a linear increase. The highest yield was recorded at 32 °C, significantly surpassing that at other temperatures, followed by a sharp decline above 34 °C (Figure 5B). Across the pH gradient, a similar unimodal response was observed. Maximal IAA production occurred at pH 7.0, while the lowest yield was recorded at pH 8.0 (Figure 5C).

### 3.7. Whole-Genome Analysis of Burkholderia pyrrocinia R74

#### 3.7.1. Basic Genomic Features of *Burkholderia pyrrocinia* R74

**Figure 6:**
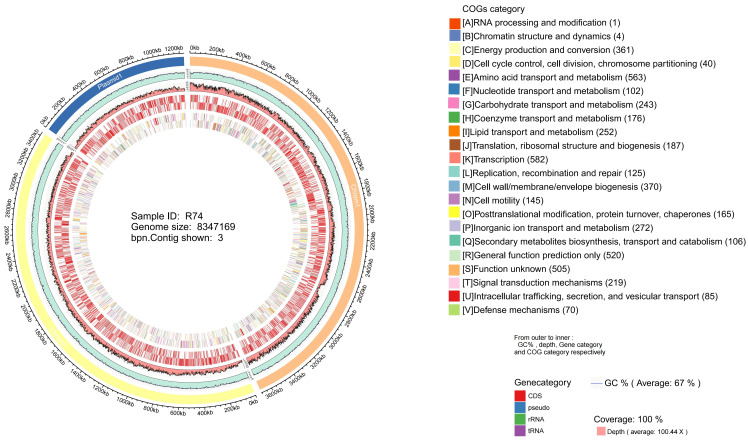
Circular genome map of strain R74. From outside to inside, the circles represent: the outermost circle (1st), genome size scale; the 2nd circle, GC content distribution; the 3rd circle, sequencing depth; the 4th and 5th circles, genetic element on the positive and negative strands, respectively; and the 6th and 7th circles, COG functional classification.

The complete genome of strain R74 is 8,347,169 bp in size and consists of two chromosomes and one plasmid, with a GC content of 67%. A total of 7543 genetic elements were predicted, including 7337 coding sequences (CDS), 18 rRNA genes, 71 tRNA genes. Additionally, 113 pseudogenes were identified, which may represent functionally degenerated or inactivated gene sequences. The genome contains 690 tandem repeats, but no significant interspersed repeats were detected, indicating high genomic stability of strain R74 (Table 2). Furthermore, 5093 genes annotated by COG were classified into 22 functional categories (Figure 6). Among these, the most abundant category was transcription, with 582 genes accounting for 11.43% of the total annotated genes. No genes related to “nuclear structure” or “cytoskeleton” were annotated, which is consistent with the genomic characteristics of prokaryotes.

#### 3.7.2. Analysis of Genes Associated with IAA Biosynthesis in Strain R74

Based on previously reported IAA biosynthesis pathways and their key enzymes [57], KEGG analysis of the R74 genome identified five enzymes potentially involved in IAA biosynthesis (Table 3). These included two key enzymes of the tryptophan side-chain oxidase (TSO) pathway, tryptophan 2,3-dioxygenase (EC 1.13.11.11) and aldehyde dehydrogenase (EC 1.2.1.3), and two key enzymes of the tryptophan-independent pathway, tryptophan synthase (EC 4.2.1.20) and indole-3-glycerol phosphate synthase (EC 4.1.1.48). Based on this, we hypothesized that the tryptophan side-chain oxidase (TSO) pathway and the tryptophan-independent pathway might serve as the primary routes for IAA biosynthesis in strain R74. In addition, the genome was found to contain amidase (EC 3.5.1.4) and aldehyde dehydrogenase (EC 1.2.1.3). Amidase catalyzed the final step of the indole-3-acetamide (IAM) pathway, converting IAM to IAA [58], whereas aldehyde dehydrogenase functioned as the terminal enzyme in the indole-3-pyruvic acid (IPyA), tryptamine (TAM), and TSO pathways, converting indole-3-acetaldehyde to IAA [59]. These findings suggested that strain R74 might also synthesize IAA via tryptophan-dependent pathways such as IAM, IPyA, and TAM pathways, although their complete pathways have not been annotated.

Based on KEGG annotation, 14 candidate genes potentially involved in IAA biosynthesis were identified in the genome of strain R74 (Table 4). Among these, five genes (Chrom1_000200, Chrom2_003794, Chrom2_003910, Chrom2_005524, and Plasmid1_006937) were predicted as key genes involved in the IAM pathway. Four genes (Chrom1_002619, Chrom2_004781, Plasmid1_006649, and Plasmid1_006891) were annotated as potential aldehyde dehydrogenase-encoding genes. Three genes (Chrom2_006360, Chrom2_006362, and Chrom1_000449) were associated with the tryptophan-independent IAA biosynthesis pathway. However, the specific functions of these candidate genes require further experimental validation.

### 3.8. Effects of Burkholderia pyrrocinia R74 on the Growth of A. spinulosa Seedlings

#### 3.8.1. Effects on Growth Parameters

**Figure 7:**
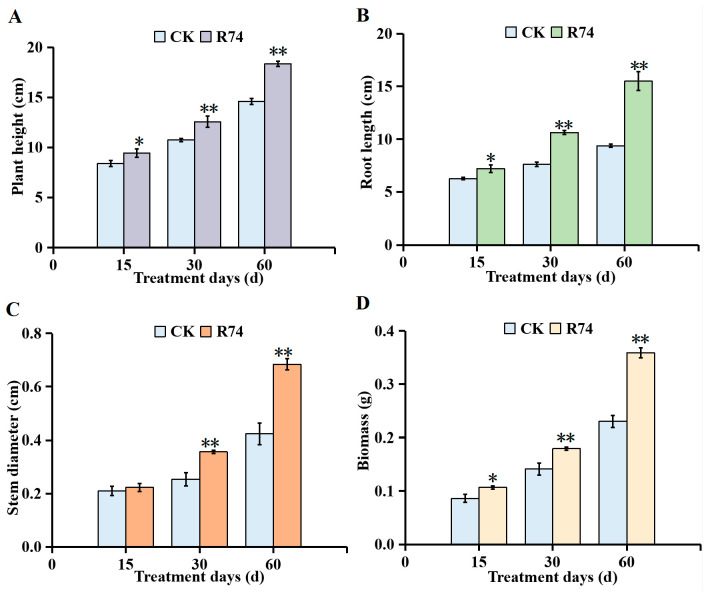
Effects of *Burkholderia pyrrocinia* R74 on the growth of *Alsophila spinulosa* seedlings: (**A**) Plant height; (**B**) Root length; (**C**) Stem diameter; (**D**) Biomass. Values are presented as mean ± SD from three replicates. Different asterisks indicate significant differences between groups based on the independent samples *t*-test: * *p* < 0.05, ** *p* < 0.01.

Inoculation with *B. pyrrocinia* R74 significantly enhanced the growth of *A. spinulosa* seedlings compared to the control group. Throughout the 60-day experimental period, plant height, root length, stem diameter, and biomass were consistently higher in R74-treated seedlings and increased progressively over time (Figure 6). At 15, 30, and 60 days post-inoculation, plant height of R74-treated seedlings increased by 12.30%, 17.08%, and 25.80%, respectively, relative to the control (Figure 7A). Root length showed corresponding increases of 14.89%, 39.30%, and 65.48% (Figure 7B). Stem diameter increased by 6.35%, 40.76%, and 61.42% at the three time points (Figure 7C), while biomass was enhanced by 23.94%, 27.12%, and 55.94% (Figure 7D).

#### 3.8.2. Effects on Chlorophyll, Soluble Protein, and Malondialdehyde Contents

**Figure 8:**
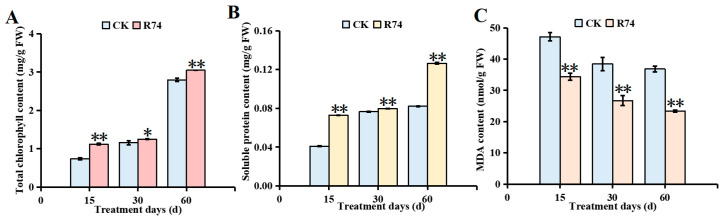
Effects of *Burkholderia pyrrocinia* R74 inoculation in rhizosphere soil on leaf physiological functions of *Alsophila spinulosa* seedlings: (**A**) Total chlorophyll content; (**B**) Soluble protein content; (**C**) Malondialdehyde (MDA) content. Values are presented as mean ± SD from three replicates. Different asterisks indicate significant differences between groups based on the independent samples *t*-test: * *p* < 0.05, ** *p* < 0.01.

Inoculation with *B. pyrrocinia* R74 significantly increased total chlorophyll content in *A. spinulosa* leaves across all sampling time points (Figure 8A). At 15, 30, and 60 days post-inoculation, chlorophyll content was elevated by 51.76%, 7.77%, and 8.93%, respectively, compared to the control group, with the highest absolute value recorded at 60 d. Most soluble proteins in plants function as enzymes involved in various metabolic processes and serve as key nutrients [60]. In this study, soluble protein content in leaves of R74-treated seedlings was markedly higher than that of the control group (Figure 8B), with increases of 78.05%, 5.26%, and 53.66% at 15, 30, and 60 days post-inoculation, respectively. These observations indicated that R74 inoculation effectively enhanced soluble protein levels in *A. spinulosa* leaves. Malondialdehyde (MDA) content reflected the degree of oxidative damage in organisms [61]. Compared to the control group, MDA content in R74-treated leaves was significantly reduced at all three time points (Figure 8C), with decreases of 57.59%, 43.93%, and 37.10% at 15, 30, and 60 days post-inoculation, respectively, which indicated that inoculation with strain R74 suppressed MDA accumulation and alleviated membrane damage in *A. spinulosa* leaves.

#### 3.8.3. Effects of *Burkholderia pyrrocinia* R74 on Antioxidant Enzyme Activities in *A. spinulosa* Leaves

**Figure 9:**
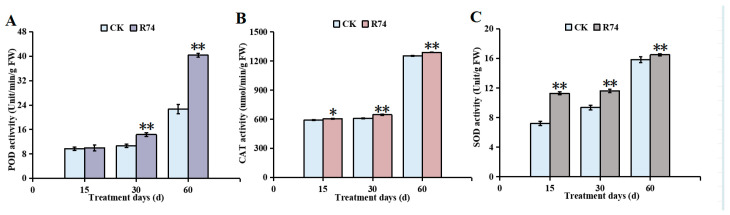
Effects of inoculating *Burkholderia pyrrocinia* R74 in rhizosphere soil on the leaf antioxidant enzyme system of *Alsophila spinulosa* seedlings: (**A**) Peroxidase (POD) activity; (**B**) Catalase (CAT) activity; (**C**) Superoxide dismutase (SOD) activity. Values are presented as mean ± SD from three replicates. Different asterisks indicate significant differences between groups based on the independent samples *t*-test: * *p* < 0.05, ** *p* < 0.01.

Inoculation with *Burkholderia pyrrocinia* R74 increased the activities of peroxidase (POD), catalase (CAT), and superoxide dismutase (SOD) in *A. spinulosa* seedlings compared to the control group. POD activity in R74-treated leaves was markedly higher than that of the control group throughout the experiment (Figure 9A). At 30 and 60 d post-inoculation, POD activity showed highly significant increases relative to the control, with the highest value recorded at 60 d (77.94% increase), indicating that R74 inoculation enhanced POD activity in *A. spinulosa*. CAT activity was also elevated in R74-treated leaves compared to the control group (Figure 9B). A significant increase was observed at 15 d, while highly significant increases were detected at 30 and 60 d post-inoculation. The maximum increase of 6.03% occurred at 30 d, confirming that R74 treatment promoted CAT activity. For SOD, highly significant increases were observed at all three time points (15, 30, and 60 d) following R74 inoculation compared to the control (Figure 9C), demonstrating that R74 effectively stimulated SOD activity in *A. spinulosa* leaves.

## 4. Discussion

Many bacterial strains isolated from plant rhizosphere soil are capable of indole-3-acetic acid (IAA) biosynthesis [62]. For instance, *Chryseobacterium aureum* D1, isolated from the rhizosphere of ancient tea forests, demonstrates pronounced IAA-biosynthetic capacity [57], and *Bacillus cereus* SZF7, screened from tea garden soils in Wuyi Mountain, also exhibits IAA-producing capacity [16]. In the present study, 39 IAA-producing bacterial strains were isolated for the first time from the rhizosphere soil of healthy *Alsophila spinulosa* plants, among which *Burkholderia pyrrocinia* R74 exhibited the highest IAA-producing capacity. The IAA production by strain R74 was 0.319 mg/L in the absence of tryptophan. Following tryptophan supplementation, its IAA yield markedly increased to 53.492 mg/(L·OD_600_). This tryptophan-dependent IAA biosynthesis characteristic is consistent with the findings of Xu et al. [63]. Although *B. pyrrocinia* JK-SH007 has been reported to produce IAA [17], its IAA yield differs from that of strain R74, potentially due to differences in strain characteristics, culture conditions, or analytical methods [64]. However, the underlying reasons require further investigation.

Culture conditions have been reported to exert a significant influence on IAA production in bacterial strains [30]. *C. aureum* achieves maximum IAA yield after 60 h of incubation at 25 °C and pH 6.0 [57], whereas *B. pyrrocinia* JK-SH007 exhibits optimal IAA production at 37 °C and pH 7.0 [17]. In the present study, strain R74 exhibited maximum IAA production under different conditions: 32 °C, pH 7.0, and 24 h of incubation. The optimal temperature and pH for IAA biosynthesis provide insights into the ecological niche occupied by bacteria within the plant rhizosphere and internal physiological environments under different temperature regimes and slightly acidic conditions [65]. These variations from previous reports may be attributed to differences in strain origin [12], although the specific underlying causes warrant further study. It should be noted that the IAA biosynthesis capacity and related metabolic characteristics reported in this study, as well as those in Molina et al. [65], were derived from in vitro culture experiments. Although these findings support our pot experiments with *A. spinulosa*, bacterial behavior under laboratory conditions may not fully reflect their actual status in the natural rhizosphere or within plant tissues. Therefore, direct extrapolation of these laboratory-based conclusions to natural field conditions has limitations, and further studies under more ecologically relevant settings are needed.

The ability of bacterial strains to produce IAA is associated with their metabolic pathways involved in IAA biosynthesis. *B. pyrrocinia* JK-SH007 synthesizes IAA via the indole-3-acetamide (IAM) pathway [17], whereas *B. pyrrocinia* LWK2 possesses only the tryptamine (TAM) pathway for IAA biosynthesis [66]. Additionally, *Variovorax boronicumulans* CGMCC4969 utilizes indole-3-acetonitrile (IAN) as a precursor for IAA production [67]. Genomic analysis of *B. pyrrocinia* R74 revealed the absence of key genes involved in the IAM, indole-3-pyruvic acid (IPyA), and TAM pathways. Instead, the strain retains key enzymes of the tryptophan side-chain oxidase (TSO) pathway, along with those associated with the tryptophan-independent pathway. This genomic architecture suggests that R74 may synthesize IAA through the TSO pathway in conjunction with the tryptophan-independent pathway, a finding consistent with observations in *Chryseobacterium* sp. D1 [57]. Notably, the intermediates of bacterial IAA biosynthesis—IAM, TAM, IAN, and IPyA—are all endogenous compounds in plants [68,69,70], raising the possibility that upon colonizing plant tissues, PGPR may utilize these plant-derived compounds as precursors for IAA production. However, how IAA biosynthesis-related genes function following PGPR colonization in plants warrants further investigation. This study revealed the plant growth-promoting characteristics of *Burkholderia pyrrocinia* strain R74, highlighting its potential as a biofertilizer. Previous studies have demonstrated that different strains of *Burkholderia pyrrocinia* possess biosafety. For example, strain S17-377 is safe for both people and plants [71], and strain JK-SH007 has been confirmed to be non-pathogenic and falls within the safe application range [72,73]. Whole-genome sequence analysis reveals that strain R74 belongs to the same species as the type strain CH-67 [74]. Accordingly, it is speculated and inferred that strain R74 also possesses good biosafety and can be further explored as a bioinoculant.

The beneficial effects of rhizosphere microorganisms on plant growth and development are well documented [75]. *Bacillus subtilis* YB-04, isolated from rhizosphere soil, promotes stem fresh weight, root fresh weight, and plant height in cucumber seedlings [76]. Inoculation with *Bacillus thuringiensis* L8 enhances fresh weight, plant height, and chlorophyll content in tobacco [77]. Similarly, *Burkholderia* sp. BK01 promotes root elongation, stem growth, and chlorophyll content in wheat seedlings [78], while *Isaria cateniannulata* increases chlorophyll content and antioxidant enzyme activities in buckwheat [79]. *Streptomyces* sp. KLBMP 5084 has also been shown to enhance both chlorophyll content and antioxidant enzyme activities in *Limonium sinense* [80]. Moreover, exogenous IAA application has been shown to increase biomass and arsenic accumulation in ferns while reducing malondialdehyde (MDA) accumulation and mitigating oxidative damage through activation of antioxidant enzyme systems [81]. In the present study, inoculation with *B. pyrrocinia* R74 significantly increased plant height, root length, stem diameter, and biomass of *A. spinulosa*, as well as soluble protein, chlorophyll, and antioxidant enzyme contents, while concomitantly reducing malondialdehyde (MDA) levels. Our results are consistent with those reported by Khan et al. [82] and Jan et al. [83], demonstrating that rhizosphere microbial application can markedly enhance plant antioxidant capacity and growth. This enhancement may be linked to improved stress resistance conferred by rhizosphere microbial inoculation, although the underlying molecular mechanisms remain to be fully explored.

## 5. Conclusions

In this study, 39 IAA-producing bacterial strains were isolated for the first time from the rhizosphere soil of *Alsophila spinulosa*, among which *Burkholderia pyrrocinia* R74 exhibited the highest IAA production. The optimal conditions for IAA production by strain R74 were determined to be 32 °C, pH 7.0, and a 24 h incubation period. Genomic analysis suggested that IAA biosynthesis in this strain occurs via the tryptophan side-chain oxidase (TSO) pathway, involving tryptophan 2,3-dioxygenase and aldehyde dehydrogenase, in conjunction with the tryptophan-independent pathway. Inoculation with strain R74 significantly enhanced the plant height, root length, stem diameter, biomass, and antioxidant capacity of *A. spinulosa*. These findings provide a theoretical foundation for further investigations into the growth-promoting mechanisms of *B. pyrrocinia* on *A. spinulosa*. It should be acknowledged that this study primarily focused on IAA biosynthesis as a potential mechanism underlying the growth-promoting effects of strain R74 and confirmed its strong IAA-producing capacity. However, PGPR may facilitate plant growth through other mechanisms such as phosphate solubilization and siderophore production, which were not systematically evaluated here. Further studies are therefore needed to fully elucidate the diverse mechanisms by which strain R74 promotes *A. spinulosa* growth.

## Figures and Tables

**Table 1:** IAA production of different strains.

Strain Number	IAA Content/mg/(L·OD_600_)	Strain Number	IAA Content/mg/(L·OD_600_)	Strain Number	IAA Content/mg/(L·OD_600_)
R3	40.010	R21	15.440	R60	22.190
R4	25.210	R22	20.810	R64	19.880
R5	28.750	R25	14.440	R67	19.780
R6	21.420	R27	18.210	R70	13.290
R9	19.030	R28	19.270	R74	53.492
R11	18.530	R31	20.130	R75	8.420
R12	16.240	R33	22.310	R76	16.750
R14	19.480	R34	14.470	R82	13.550
R15	22.030	R37	27.140	R83	7.330
R16	16.690	R38	16.470	R90	14.200
R17	20.320	R41	17.250	R92	9.020
R19	18.120	R43	15.540	R93	20.340
R20	12.320	R53	16.370	R97	11.220

**Table 2:** Basic characteristics of the genome of *Burkholderia pyrrocinia* strain R74.

Features	Chromosome
Total size of the contigs (bp)	8,347,169
GC (%)	67%
N50 (bp)	3,428,300
Total Contigs	3
Total genes	7543
Number of CDS	7337
Number of tRNA genes	71
Number of rRNA genes	18
Number of ncRNA genes	4
Pseudo-gene	113
Total TRF	690

**Table 3:** Functional annotation in the IAA biosynthetic pathway based on KEGG database.

Pathway	Enzyme	Strain R74
Indole-3-acetamide (IAM)	Trypthophan 2-monooxygenase (EC: 1.13.12.3)	−
	Amidase (EC: 3.5.1.4)	+
Indole-3-pyruvic acid (IPyA)	Trp aminotransferase (EC: 2.6.1.27)	−
	Indolepyruvate decarboxylase (EC: 4.1.1.74)	−
	Aldehyde dehydrogenase (EC: 1.2.1.3)	+
Indole-3-acetonitrile (IAN)	Trp-2-monoxigenase (EC: 1.13.12.3)	−
	Nitrile hidratase (EC: 4.2.1.84)	−
	Nitrilase (EC: 3.5.5.1)	−
Tryptamine (TAM)	Trp decarboxylase (EC: 4.1.1.28)	−
	Monoamine oxidase (EC: 1.4.3.4)	−
	Aldehyde dehydrogenase (EC: 1.2.1.3)	+
Tryptophan Side-Chain Oxidase (TSO)	Tryptophan-2,3-dioxygenase (EC 1.13.11.11)	+
	Aldehyde dehydrogenase (EC1.2.1.3)	+
Tryptophan-Independent	Tryptophan synthase (EC 4.2.1.20)	+
	Indole-3-glycerol phosphate synthase (EC 4.1.1.48)	+

“+” represents that the gene has been identified; “−” represents unrecognized gene.

**Table 4:** Information on candidate genes involved in IAA production.

Gene	Start	End	Strand	Protein Sequence Length/aa	Nucleotide Sequence Length/bp	Description
Chrom1_000200	229,392	230,871	−	492	1479	IAM
Chrom2_003794	449,126	450,251	+	374	1125	IAM
Chrom2_003910	578,656	580,057	−	466	1401	IAM
Chrom2_005524	2,352,250	2,353,735	+	494	1485	IAM
Plasmid1_006937	540,345	541,761	−	471	1416	IAM
Chrom1_002619	2,829,877	2,831,317	+	479	1440	IPyA/TAM/TSO
Chrom2_004781	1,519,488	1,521,000	+	503	1512	IPyA/TAM/TSO
Plasmid1_006649	227,321	228,803	−	493	1482	IPyA/TAM/TSO
Plasmid1_006891	487,634	489,074	−	479	1440	IPyA/TAM/TSO
Chrom1_001307	1,407,631	1,408,573	+	313	942	TSO
Chrom1_002791	3,009,268	3,010,204	+	311	936	TSO
Chrom2_006360	3,307,473	3,308,289	−	271	816	Tryptophan- Independent
Chrom2_006362	3,309,191	3,310,385	−	397	1194	Tryptophan- Independent
Chrom1_000449	480,112	480,898	−	261	786	Tryptophan- Independent

“+” indicates the forward strand; “−” indicates the reverse strand.

## Data Availability

The original contributions presented in this study are included in the article/Supplementary Materials. Further inquiries can be directed to the corresponding authors. The raw sequencing reads of this strain were deposited in the NCBI SRA database under accession numbers SRR38081102 and SRR38081103. The whole-genome sequencing project was registered in GenBank with BioProject accession PRJNA1452564 and BioSample accession SAMN57238736. All relevant genomic data are publicly available in the NCBI database.

## Supplementary Materials

The following supporting information can be downloaded at: https://www.mdpi.com/article/10.3390/microorganisms14051103/s1, Figure S1: Qualitative determination of the ability of bacterial strains to produce indole-3-acetic acid.
