# Supplementary material for: Isolation, Identification, and Functional Characterization of a Rhizosphere Bacterium Promoting the Growth of Alsophila spinulosa
Source: Microorganisms. 2026 May 13;14(5):1103. doi: 10.3390/microorganisms14051103 (PMC13210164; doi:10.3390/microorganisms14051103)
Supplement: Supplementary file 1 [file microorganisms-14-01103-s001.zip › microorganisms-4219687-supplementary.pdf]

Supplementary Materials S1

This file contains supplementary figures related to the manuscript, which provide additional data supporting the results and discussion in the main text. All supplementary figures are referenced in the manuscript.

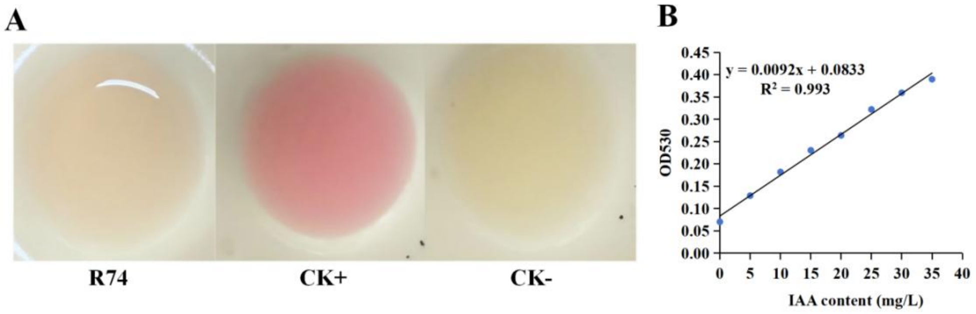

Figure S1. Qualitative determination of the ability of bacterial strains to produce indole-3-acetic acid. CK−: Negative control, LB medium; CK+: Positive control, IAA standard solution.
